# Social cognition in borderline personality disorder

**DOI:** 10.3389/fnins.2012.00195

**Published:** 2013-01-14

**Authors:** Stefan Roepke, Aline Vater, Sandra Preißler, Hauke R. Heekeren, Isabel Dziobek

**Affiliations:** ^1^Department of Psychiatry, Charité - Universitätsmedizin Berlin, Campus Benjamin FranklinBerlin, Germany; ^2^Freie Universität Berlin, Cluster of Excellence Languages of EmotionBerlin, Germany; ^3^Department of Biological und Clinical Psychology, Friedrich Schiller University Jena UniversityJena, Germany

**Keywords:** borderline personality disorder, social cognition, empathy, affective instability, posttraumatic stress disorder

## Abstract

Many typical symptoms of borderline personality disorder (BPD) occur within interpersonal contexts, suggesting that BPD is characterized by aberrant social cognition. While research consistently shows that BPD patients have biases in mental state attribution (e.g., evaluate others as malevolent), the research focusing on accuracy in inferring mental states (i.e., cognitive empathy) is less consistent. For complex and ecologically valid tasks in particular, emerging evidence suggests that individuals with BPD have impairments in the attribution of emotions, thoughts, and intentions of others (e.g., Preißler et al., 2010). A history of childhood trauma and co-morbid PTSD seem to be strong additional predictors for cognitive empathy deficits. Together with reduced emotional empathy and aberrant sending of social signals (e.g., expression of mixed and hard-to-read emotions), the deficits in mental state attribution might contribute to behavioral problems in BPD. Given the importance of social cognition on the part of both the sender and the recipient in maintaining interpersonal relationships and therapeutic alliance, these impairments deserve more attention.

## Introduction

Borderline Personality Disorder (BPD) is a severe psychiatric condition characterized by a pervasive pattern of marked impulsivity and instability in affects, self-image and interpersonal relationships (APA, 2000). BPD affects 1–3% of the general adult population (Trull et al., 2010) and 10% of psychiatric out-patients, as well as 20% of in-patients (Korzekwa et al., 2008). BPD is therefore a prominent clinical disorder in psychiatric contexts.

Empirical research on BPD has thus far mostly focused on **affective instability**. Although affective instability has been well established as a core symptom of BPD, it is not specific to the disorder and does not solely explain it, as it is a common characteristic in other psychiatric conditions [e.g., depressive- and bipolar-spectrum conditions, and posttraumatic stress disorder (PTSD), see Koenigsberg, 2010]. There are a number of prominent symptoms of BPD—including repetitive suicidal behavior, self-injury, aggressive outbursts, and increased emotional reactivity—that typically manifest themselves in an interpersonal context. This supports the idea of a superordinate deficit in the perception, processing and emission of **social signals** (Stiglmayr et al., 2005; Brodsky et al., 2006; Gunderson and Lyons-Ruth, 2008).

Although disturbed interpersonal functioning has been acknowledged since the early descriptions of BPD (Stern, 1938; Kernberg, 1967), it is only within the last decade that research has focused more closely on the behavioral and neural underpinnings of this aspect of the disorder (e.g., Hill et al., 2008; King-Casas et al., 2008; Seres et al., 2009; Ruocco et al., 2010) and now supports the notion that the relational style of BPD patients might be more specific to the disorder than affective instability or impulsivity (Gunderson, 2007). The exchange of social signals that is the basis for **social cognition** could be a key factor in understanding the characteristic relational style in BPD. This article aims to review the current status of research on the processes of social cognition in patients with BPD.

## Social cognition

Social interaction involves bi-directional processes: individuals are emitters and recipients of social signals. The function of adequately perceiving and processing social signals (consciously or unconsciously) has been referred to as social cognition (c.f., Adolphs, 1999; Frith and Frith, 2007). The outcome of this process depends upon the interpretation of social signals emitted during the encounter, including not only language (content and tone), but also facial expression and body gestures. Moreover, the ability to adequately process social signals is a prerequisite for consciously or unconsciously generating appropriate responses. Thus, social cognitive skills are necessary for successful social interactions and they allow humans to establish and maintain, short- and long-term relationships with significant others.

A construct that captures a wide range of social cognition processes is empathy. There is broad agreement that empathy comprises at least two components (Singer, 2006; Decety and Meyer, 2008). The first is a cognitive component, which captures the capacity to infer others' mental states and is also referred to as perspective taking, mentalizing, or (affective and cognitive) theory of mind (Blair, 2005). Second, empathy also comprises an affective component, i.e., an emotional response to another person's emotional state (Davis, 1994; Blair, 2005).

In the following, we review literature that provides data on BPD patients as recipients and emitters of social signals. With regard to BPD patients as recipients of social signals, we focus on the ability for **cognitive empathy** and **emotional empathy**. With respect to BPD patients as emitters of social signals, we focus on studies of facial emotion expression. In addition, we present new approaches that use economic exchange games to mimic the bi-directional process of social exchange (e.g., King-Casas et al., 2008).

## Cognitive empathy in BPD

A variety of different study designs have been utilized to assess cognitive empathy in BPD. These studies have focused on accuracy or biases of mental state attribution, using self- and other reports, as well as behavioral and neuroimaging tasks.

Early psychoanalytic studies used Rorschach responses to investigate general ability and biases in cognitive empathy in BPD patients (e.g., Lerner and St. Peter, 1984; Stuart et al., 1990; for review see Westen, 1990). In summary, borderline patients showed more malevolent and idiosyncratic, yet cognitive-developmentally advanced representations of people's intentions on the Rorschach test. Additional research on biases using projective material from the Thematic Apperception Test and other narratives (Westen, 1990, 1991a,b; Westen et al., 1990a,b,c,d; Nigg et al., 1992; Segal et al., 1992, 1993) further indicated that BPD patients were able to make complex intentional attributions of other people's actions. However, BPD patients expressed more malevolent representations of others compared to the control groups of non-BPD patients and non-clinical participants. A study by Arntz and Veen (2001), however, found evidence for less complexity in BPD patients', but also cluster C personality disorder patients', character descriptions after watching emotional and non-emotional film sequences compared to non-clinical controls. A further study (Veen and Arntz, 2000) used short video clips from commercial movies to assess biases and found that BPD patients (compared to cluster C personality disorder patients and non-clinical controls) made more extreme evaluations (i.e., multidimensional dichotomous thinking) of actors in film clips addressing BPD specific themes, but not in control film clips. Another study by the same group (Arntz et al., 2012) evaluated telephone discussions on real negative events of BPD patients and control groups, with professionals taking an accepting, rejecting or neutral stance. BPD patients showed no significant difference in complexity of understanding of others but displayed a more extreme evaluation (i.e., multidimensional dichotomous thinking) of the professionals in all three conditions compared to cluster C personality disorder patients and non-clinical participants. Further, Barnow et al. (2009) conducted a study on interpretation biases by presenting short silent film clips of characters entering a room and taking a seat. BPD patients evaluated the characters in the film as more negative and more aggressive compared to depressed and non-clinical controls. Furthermore, studies using self-report questionnaires to assess biases found that BPD patients had a tendency to assume that “the world and others are dangerous and malevolent” (Pretzer, 1990; Arntz et al., 2004).

Using self-report measures on ability of cognitive empathy, Guttman and Laporte (2000) found perspective taking, as assessed with the Interpersonal Reactivity Index (IRI; Davis, 1983), to be impaired in BPD patients compared to patients with anorexia nervosa and non-clinical controls. Harari et al. (2010) and New et al. (2012) replicated this finding on the IRI perspective taking scale in BPD patients compared to non-clinical controls. Fonagy et al. (1996) used a clinical interview in the style of the Adult Attachment Interview (de Haas et al., 1994) and found further evidence that BPD patients had deficits in understanding the mental states of others compared to a clinical non-BPD control group.

Clinical observations (Krohn, 1974; Carter and Rinsley, 1977) and early empirical studies focusing on accuracy in inferring others' emotional states (Frank and Hoffman, 1986; Ladisich and Feil, 1988) gave rise to the descriptive term “borderline empathy”, which refers to enhanced cognitive empathy in BPD. A study by Frank and Hoffman (1986) analyzed the ability of BPD patients to infer the emotional states of others compared to non-BPD patients. Participants had to choose one of two alternative affective descriptions after watching a 10-min video sequence containing depictions of different emotional situations, each portrayed by the same female actor. The borderline group showed significantly fewer errors and was more sensitive to nonverbal communication than the control group, thus indicating increased cognitive empathy in BPD. Ladisich and Feil (1988) measured how well a particular member of an interacting group predicted the self-rated feelings of the other group members. BPD patients achieved higher scores compared to non-BPD patients but did not differ from the psychiatrist's ratings, which served as further argument for increased cognitive empathy in BPD. Flury et al. (2008) used a comparable study design including participants with high and low BPD traits. In the first step, the authors replicated the results of Ladisich and Feil (1988), with individuals with high BPD traits showing enhanced accuracy in attributing mental states (thoughts and feelings) to others. In a second step, however, reanalysis of the data revealed that these effects were a consequence of the participants with high BPD traits having more unusual, harder-to-predict personalities, and thoughts and feelings that were difficult to infer compared to their counterparts with low BPD traits. This led to lower accuracy scores in the participants with low BPD traits (Flury et al., 2008). The authors concluded that the difference in accuracy between individuals with low and high BPD traits was not related to a difference in performance but to the difficulty in reading high BPD trait participants. Thus, this study presented a first hint that emission of social signals might be abnormal in BPD.

Most recent studies on accuracy in cognitive empathy in BPD have used facial emotion recognition tasks (e.g., by using static images, such as Ekman faces or morphed facial pictures; Lynch et al., 2006; Domes et al., 2008; for review see Domes et al., 2009). The results of these studies have not been entirely consistent. In some studies, patients with BPD correctly identified emotional facial expressions (Wagner and Linehan, 1999), at times even more accurately than non-clinical controls (Lynch et al., 2006). Although Wagner and Linehan (1999) found that neutral facial expressions were interpreted more negatively by BPD patients compared to non-clinical controls. Domes et al. (2008) examined the ratings of pictures of faces displaying two basic emotions at the same time (i.e., blends). BPD patients showed a bias toward the perception of anger in comparison to non-clinical controls. Interestingly, when facial emotion recognition tasks approximate more complex and naturalistic situations [e.g., by setting time limits for recognizing emotions in faces (Dyck et al., 2009), or by providing additional prosodic information (Minzenberg et al., 2006)], patients with BPD show increased error rates compared to non-clinical controls. Thus, these findings might indicate that BPD patients show impairments in cognitive empathy mainly on tasks that are complex or more ecologically valid.

In a study using the “Reading the Mind in the Eyes” (RME; Baron-Cohen et al., 2001) test that focuses on accuracy of inferring emotional states by presenting photographs of eye regions, Schilling et al. (2012) could not detect any deficits in mental state attribution in BPD patients compared to non-clinical controls. In contrast, Fertuck et al. (2009) found enhanced “mindreading” capacities with the RME test in BPD patients compared to non-clinical controls.

Using the “Faux pas” task that focuses on accuracy of inferring thoughts and intentions, Harari et al. (2010) found theory of mind to be impaired in BPD patients compared to non-clinical controls. In the “Faux pas” task (Baron-Cohen et al., 1997) 20 stories with interactions between speaker and listener are presented and the participant is asked to detect a faux pas in each story. Arntz et al. (2009) also focused on accuracy in mental state inferences and could not find deficits in theory of mind capacities in BPD patients compared to non-clinical controls when using the “strange stories” task; however, BPD patients scored significantly lower than cluster C personality disorder patients. The “strange stories” task (an advanced Theory of Mind task, Happé, 1994) includes stories involving bluffs, mistakes, white lies, and persuasion in addition to non-mental state stories as a control condition. After listening to the stories, participants are asked questions about the characters' intentions. Furthermore, Ghiassi et al. (2010) did not find deficits in understanding others' minds in BPD patients compared to non-clinical controls in a study using a cartoon task that also focuses on accurately inferring thoughts and intentions. In the cartoon task (Brüne, 2005), cartoon pictures with stories of cooperation, cheating, and cooperation at the cost of a third person have to be ordered in a logical sequence.

In sum, studies on accuracy in cognitive empathy in BPD have not produced consistent results. One limitation of prior study designs is the lack of ecological validity, given that BPD patients only seem to show deficits in cognitive empathy in tasks using more complex or ecologically valid material (Minzenberg et al., 2006; Dyck et al., 2009).

In response to the critique of stimulus material with low ecological validity, Dziobek and colleagues developed the “Movie for the Assessment of Social Cognition” (MASC; Dziobek et al., 2006; Hassenstab et al., 2007). The MASC is video-based and displays social interactions among multiple characters, thus including social signals such as language, gesture, posture, and facial expression. It assesses the participant's recognition of the characters' mental states in an everyday life context and thus allows for the analysis of cognitive empathy in a more ecologically valid manner than traditional tests. In the movie, four people spend an evening together having dinner. Dominant interaction topics include dating and friendship. Given that patients with BPD show defining social abnormalities with respect to friendship and romantic relationships, the MASC might be specifically sensitive to social cognitive dysfunctions. The 15-min film is paused at 45 points when questions concerning the characters' feelings, thoughts, and intentions are asked (e.g., “What is Betty feeling?,” “What is Cliff thinking?,” and “Why is Michael doing this?”).

A recent study by Preißler et al., (2010) assessed cognitive empathy using the MASC and the RME task in 64 women diagnosed with BPD, and 38 non-clinical female comparison subjects matched in age and IQ. Whereas the RME task failed to detect significant impairments in social cognition in patients with BPD, the more ecologically valid MASC clearly identified differences. Patients with BPD showed impaired recognition of the feelings, thoughts, and intentions of the starring movie characters. The results of this study support the notion that BPD patients have deficits in cognitive empathy in more ecologically valid tasks. A limitation of this study might be that the MASC focuses on dating and friendship issues. Thus, it cannot be ruled out that this deficit is context specific and does not apply to other social situations.

In another study, applying the MASC in adolescents with BPD traits, Sharp et al. (2011) also found evidence for impaired cognitive empathy in individuals high in borderline traits in comparison to individuals low in borderline traits. Moreover, cognitive empathy correlated with self-report measures of emotion regulation. These results indicate that high arousal or emotional states might interfere with cognitive empathy ability (Sharp et al., 2011).

Another study by Dziobek et al. (2011) further assessed cognitive empathy in BPD using the Multifaceted Empathy Test (MET; Dziobek et al., 2008). The MET is considered a more ecologically valid measure than self-report instruments or text-based tasks, and it has the additional benefit of allowing for the separate assessment of cognitive and emotional aspects of empathy. The test consists of photographs depicting people in emotionally charged situations and is intended to produce strong emotional reactions. To assess cognitive empathy, participants were required to infer the emotion of the subject in the photo and were asked to indicate the correct one from a list of four. This study replicated the finding of a deficit in cognitive empathy in patients with BPD (Dziobek et al., 2011).

### Impact of childhood maltreatment and trauma on cognitive empathy in BPD

Prior research has indicated that childhood maltreatment has a negative impact on different aspects of social cognition in non-clinical individuals (Smith and Walden, 1999; Cicchetti et al., 2003; Pears and Fisher, 2005).

Although Ghiassi et al. (2010) did not find that BPD patients had deficits in understanding others' minds using a cartoon task, self-reported negative maternal behavior was a negative predictor for cognitive empathy later in adulthood, indicating the negative influence of early stressors.

Furthermore, the results by Preißler et al., (2010) provided preliminary evidence that sexual assault by a known assailant, which was reported by 58% of patients, is associated with impaired mental state attribution. These findings are of special interest as BPD is associated with high rates of childhood maltreatment (Zanarini, 2000). Physical abuse or neglect, and especially sexual abuse are specific environmental risk factors for developing BPD (Johnson et al., 1999; Lobbestael et al., 2010). Furthermore, BPD patients report more types of abuse in childhood, beginning earlier in life and repeated over longer periods of time than comparison groups (Zanarini et al., 1997). The study by Preißler et al., (2010) found that only 46% of BPD patients who reported sexual assault by a known assailant developed comorbid PTSD. Thus, this type of trauma seems to be associated with impairment in cognitive empathy in BPD patients even in the absence of the full clinical picture of comorbid PTSD.

At the same time, PTSD is a prevalent comorbidity of BPD as it is present in up to 56% of patients (e.g., Zanarini et al., 1998). This high percentage of full symptom PTSD, but also the frequent subclinical PTSD (Harned et al., 2010) is reflected in, among other symptoms, the severe intrusions that many BPD patients suffer from. Preliminary evidence from studies using facial emotion recognition tasks showed that intrusive symptoms and comorbid PTSD are negative predictors for emotion recognition abilities in BPD (Dyck et al., 2009). Preißler et al., (2010) also identified intrusive symptoms and comorbid PTSD in their study as factors contributing to impaired cognitive empathy in BPD. However, further studies are needed to assess more precisely the impact of trauma, trauma type and comorbid PTSD on cognitive empathy in BPD and, most importantly, to identify underlying mechanisms.

In sum, studies on bias in cognitive empathy in BPD point toward a generally differentiated but negative and malevolent perception of others, especially if borderline-specific themes are addressed (e.g., rejection, exclusion, and abuse). Further, BPD patients might be characterized by a bias toward a generally more extreme evaluation of others. Studies using self-report measures indicate that BPD patients themselves perceive a deficit in cognitive empathy. Studies on accuracy of cognitive empathy in BPD seem to indicate an impaired ability, especially in patients with comorbid PTSD or a history of sexual abuse. These deficits in accuracy are subtle, might depend on context, and are more likely to be detected with ecologically valid tasks.

### Neurofunctional correlates of cognitive empathy in BPD

Recent data have provided initial evidence for alterations in brain function that might underlie deficits in cognitive empathy in BPD. Dziobek et al. (2011) used a functional Magnetic Resonance Imaging (fMRI) adaptation of the MET (Dziobek et al., 2008) and compared 30 un-medicated women with BPD and 29 female non-clinical controls. Brain responses during cognitive empathy were significantly reduced in BPD patients compared to controls in a region comprising the left superior temporal sulcus and gyrus (STS/STG). In line with these findings, a recent study by Mier et al. (2012) also found reduced activation of the STS/STG along with the inferior frontal gyrus during the attribution of intentions compared to non-clinical controls in a cognitive empathy task. The STS/STG is known for its role in social cognition and is an important part of the neural network that mediates thinking about others (Saxe and Kanwisher, 2003; Bahnemann et al., 2010). Interestingly, reduction of STS/STG activation in the study by Dziobek et al. (2011) was associated with levels of intrusive symptomatology in the BPD group: those individuals showing particularly low levels of activation in the STS/STG region reported high levels of recurring traumatic memories. Interestingly, the STS region matures late in ontogeny (Paus, 2005), rendering it particularly vulnerable to on-going early psychosocial stressors. Thus, one might speculate that childhood maltreatment has an impact on the developing brain, which might result in STS/STG dysfunction. Growing up in a malevolent social environment might hinder adequate learning experience that is necessary for acquiring cognitive empathy capacities.

## Emotional empathy in BPD

The construct of emotional empathy (Mehrabian and Epstein, 1972; Eisenberg and Miller, 1987) describes an observer's emotional response to another person's emotional state. Only a few attempts have been made to assess emotional empathy in BPD. Harari et al. (2010) used the IRI (Davis, 1983) and found that self-reported affective aspects of empathy (empathic concern) were slightly increased among BPD patients compared to non-clinical controls. New et al. (2012), however, did not find significant group differences between BPD patients and non-clinical controls using the same measure. In contrast, Dziobek et al. (2011) found a trend toward decreased values in BPD using the IRI empathic concern scale. Dziobek et al. (2011) additionally used the more ecologically valid MET to assess emotional empathy. In the emotional empathy items of the MET, participants were required to rate the amount of mirroring of an emotion that took place in response to a picture (e.g., if the mental state of the person was anxious, subjects were asked to rate how anxious they felt) and additionally rated the degree of empathic concern they felt for the person in the picture. Results from the MET revealed that BPD patients had significantly reduced tendencies to feel empathy for other people in emotionally distressing situations compared to non-clinical controls (Dziobek et al., 2011).

### Neurofunctional correlates of emotional empathy in BPD

Applying the fMRI version of the MET, Dziobek et al. (2011) found that during emotional empathy the right mid-insula was more activated in individuals with BPD than in non-clinical controls. The mid-insula has been shown to react strongly to bodily states of arousal (Brendel et al., 2005). Further, Dziobek et al. (2011) found a positive association between the activation in the right middle insula and skin conductance during emotional empathy in individuals with BPD, which supports the notion of increased arousal when being emotionally involved with others. Higher levels of personal distress and arousal are commonly observed in individuals with BPD (e.g., Guttman and Laporte, 2000). Thus, the data of Dziobek et al. (2011) suggest that arousal might interfere with emotional empathy in BPD. Other studies with non-clinical samples support this assumption by showing that individuals who are able to regulate their emotions are more likely to experience concern for others (Eisenberg et al., 1994). Thus, the deficit in the ability to regulate emotions in BPD might be directly linked to impairments in emotional empathy.

## Facial emotion expression in BPD

BPD patients are not only recipients but also emitters of social signals. The emission of unclear or hard to read social signals might contribute to social dysfunction in BPD. Among these signals, facial expressions play a major role in communication (Frith, 2009). Even unconscious perceptions of facial emotional expressions can lead to behavioral and emotional contagion in the observer. These perceptions might therefore act as one very basal mechanism for inferring the mental state of an interaction partner (Dimberg et al., 2000; Frith, 2009).

To date, only a small number of empirical studies have examined altered nonverbal expression in BPD. As illustrated above, Flury et al. (2008) assessed empathic accuracy in interacting dyads. Their results indicated that thoughts and feelings of students with high BPD features are harder to infer compared to their counterparts with low BPD features. Moreover, previous studies have specifically examined facial emotional expressions in patients with BPD (Herpertz et al., 2001; Renneberg et al., 2005). In one study, male criminal offenders with BPD showed little facial response to pleasant and unpleasant stimuli compared to non-clinical controls (Herpertz et al., 2001). In another study, frequency and intensity of facial emotional expressions in female patients with BPD were assessed while participants watched film sequences of positive or negative emotional valence (Renneberg et al., 2005). In line with the results of Herpertz et al. (2001), patients with BPD reacted in the same manner as depressed patients, with reduced facial activity compared to non-clinical controls. A further argument supporting reduced facial activity comes from a study by Lobbestael and Arntz (2010), which found reduced facial response in BPD patients after presentation of abuse-related film stimuli compared to non-clinical and antisocial personality disorder control groups.

In a recent study, Staebler et al. (2011) used a social exclusion paradigm to study facial emotion expression in BPD. In a virtual ball tossing game (Cyberball, for review see Williams, 2007) that has been shown to induce strong emotional reactivity in BPD (Renneberg et al., 2012), facial emotion expressions were analyzed applying the Emotional Facial Action Coding System (EMFACS; Ekman et al., 1994). The results revealed that BPD patients reacted with fewer positive expressions and with more mixed emotional expressions (two emotional facial expressions at the same time, e.g., anger and sadness) compared to a non-clinical control group when socially excluded in the ball tossing game. That being said, shame—which is a frequent emotion in BPD (Rüsch et al., 2007) and might likely have been elicited in the exclusion situations—was not assessed in the study. EMFACS does not allow for the measurement of shame, given that no distinct facial expression has been described for this complex emotion (c.f., Tracy and Robins, 2004). This might have confounded the assessment of mixed emotions. Thus, future research needs more fine-grained measurement of mixed basic emotions while also accounting for complex self-conscious emotions such as shame. Nevertheless, the results indicate that nonverbal signs of facial emotion expression are deviant in BPD patients compared to controls. This could play an important role in the disturbed social relationships of patients with BPD, given that deviant facial expressions represent unreliable sources for mental state decoding on the part of their interaction partners.

## An integrative framework for social cognition in BPD

With regard to the reception of social signs in individuals with BPD, studies have focused primarily on accuracy or bias in cognitive empathy. Research on bias in cognitive empathy showed that although BPD patients generally are able to make complex intentional attributions about others, they show systematic negative, malevolent biases. In addition, it was suggested that their evaluation of others is generally more extreme. Whereas earlier work on accuracy has found no deficits in cognitive empathy, more recent work using more ecologically valid and complex stimuli has shown that BPD patients have subtle deficits in the ability to infer the emotions, thoughts, and intentions of others (Minzenberg et al., 2006; Dyck et al., 2009; Preißler et al., 2010; Dziobek et al., 2011). High arousal might additionally interfere with BPD patients' ability for cognitive empathy (Sharp et al., 2011). Furthermore, comorbid PTSD and intrusive symptomatology are related to deficits in cognitive empathy in BPD (Preißler et al., 2010). Neurofunctionally, these deficits are associated with reduced activity in important nodes of the cognitive empathy network, i.e., the STS/STG region and the inferior frontal gyrus (Dziobek et al., 2011; Mier et al., 2012). Interestingly, reduced STS/STG activation in BPD was shown to predict PTSD symptomatology (Dziobek et al., 2011). PTSD is caused by the experience of traumatic events. Preliminary data indicate that trauma such as sexual abuse by a known assailant is an additional independent negative predictor for cognitive empathy in BPD (Preißler et al., 2010). Thus, these data suggest that experience of traumatic events and subsequent PTSD worsen cognitive empathy capacities. One can speculate that a negative, traumatic learning environment hinders the formation of fully developed cognitive empathy.

BPD involves subtle deficits in the appropriate emotional reaction to another person, i.e., emotional empathy (Davis, 1994). Whereas with more ecologically valid tasks these impairments were detectable (Dziobek et al., 2011), this was not the case with less ecologically valid questionnaires (Harari et al., 2010; Dziobek et al., 2011; New et al., 2012). Neurofunctionally, increased activation of the medial insula (which negatively correlates with changes in skin conductance during an emotional empathy task) suggests that arousal or distress might interfere with the capacity of emotional empathy in BPD (Dziobek et al., 2011).

As senders of social signals, BPD patients show deviant facial emotional reactions to social stimuli (Herpertz et al., 2001; Renneberg et al., 2005). Particularly, aversive social stimuli and contexts such as social exclusion evoke hard-to-read, mixed facial emotion expressions in BPD (Staebler et al., 2011).

**Figure 1:**
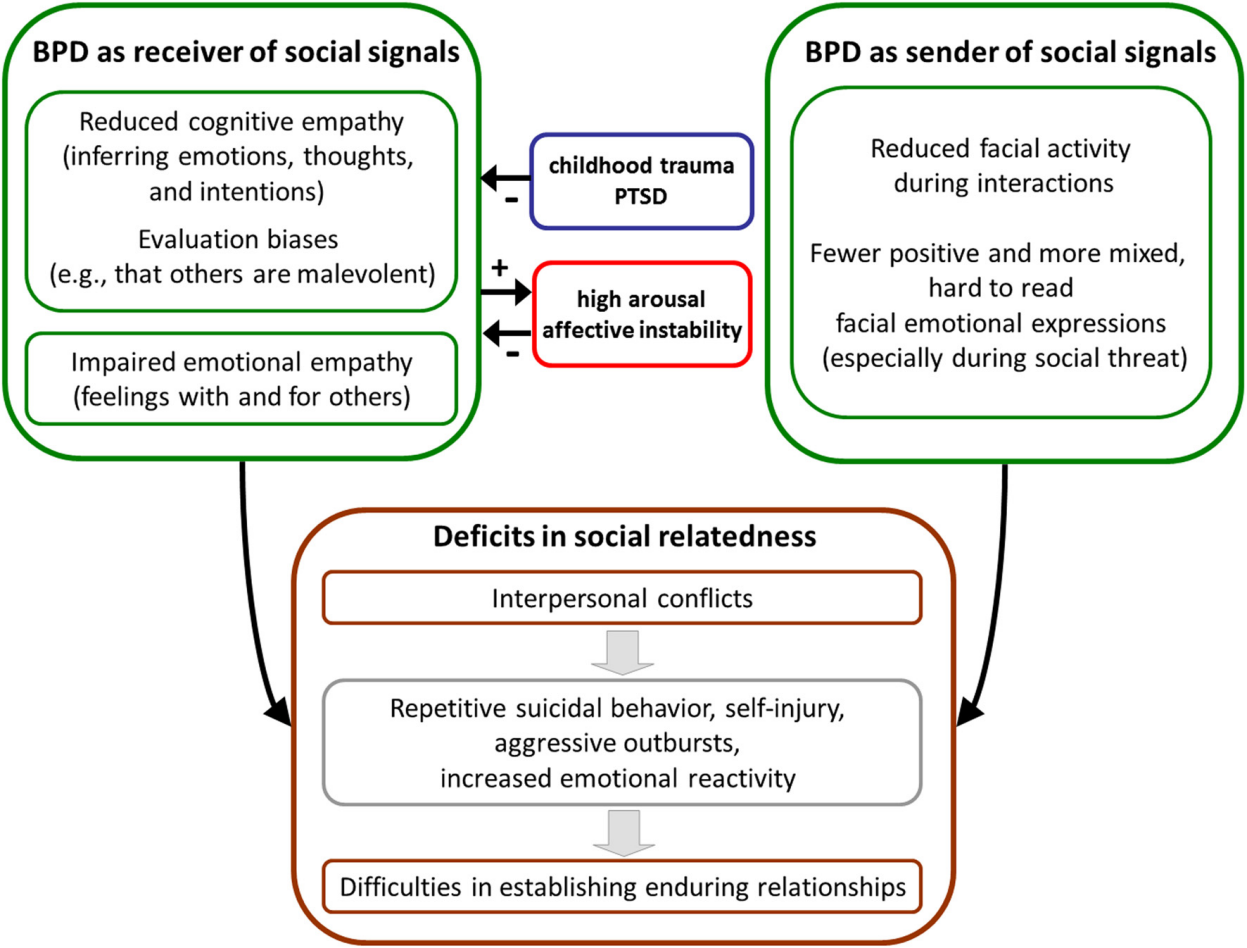
**Possible links between deviant social cognition and deficits in social relatedness in borderline personality disorder (BPD)**.

Aberrant perception and understanding of social signals as well as emission of ambiguous, hard-to-read social signals might significantly contribute to unstable interpersonal relationships and other related psychopathological features in BPD that occur in the context of social settings. One underlying mechanism seems to be that BPD-specific processing of social signals results in increased emotional reactivity (Koenigsberg, 2010). This in turn might further impair cognitive, as well as affective, empathy in BPD (Dziobek et al., 2011; Sharp et al., 2011). Together with aberrant facial expressions, the depicted vicious cycle might lead to interpersonal conflicts that provoke aggressive outbursts, repetitive suicidal behavior, self-injury, and other BPD-typical behavior (e.g., Gunderson and Lyons-Ruth, 2008). As a consequence, the sum of those processes might lead to impairments in establishing effective social interactions, provoke repetitive interpersonal conflicts with significant others, and lead to difficulties in establishing stable long-term relationships in BPD (Figure 1).

## Future directions

Deficits in social cognition have been described in a number of psychiatric disorders (e.g., euthymic bipolar patients; Montag et al., 2010; autism spectrum conditions, Dziobek et al., 2006; narcissistic personality disorder; Ritter et al., 2011). It will be an important task for future research to characterize the specific aspects of dysfunctional social cognition abilities unique to BPD by, for example, using comparative study designs including other disorders with social dysfunction.

The data we reviewed highlight several social cognitive impairments in BPD, which are modulated by personal distress and arousal. To provide evidence for the specificity of those findings, further studies need to assess social cognition under different emotional conditions. Prior research indicates that although BPD patients might not be more physiologically reactive to emotional cues in general, they might show increased emotional vulnerability if borderline-specific themes are addressed and comorbid PTSD is present (Limberg et al., 2011). In particular stimuli inducing perception of rejection or abandonment are able to elicit arousal and strong negative emotions such as anger and rage in BPD (Berenson et al., 2011; Limberg et al., 2011; Renneberg et al., 2012). Therefore, future studies should use these stimuli to assess social cognition under varying arousal and emotional conditions and in different social contexts.

To date, research on cognitive empathy has focused on either bias or accuracy. In the future, study designs should combine both approaches to gain an integrated understanding of mental state attribution in BPD. Further, emerging evidence in non-clinical groups has shown that cognitive empathy depends on motivation (Ickes, 2011), that is either externally induced and thus context-dependent (e.g., attractiveness of the encounter) or related to personality characteristics or personality pathology. Additionally, recent research suggests that non-clinical participants have a well-calibrated understanding of when they are accurate in inferring mental states (Kelly and Metcalfe, 2011). Thus, assessing metacognitive awareness of emotional and cognitive empathy might be relevant to BPD research. Finally, BPD patients present with high levels of alexithymia. This associated feature of BPD might have an impact on emotional and cognitive empathy (Guttman and Laporte, 2002; New et al., 2012), which should be accounted for in future study designs.

Most previous studies of social cognition in BPD used unidirectional tests (e.g., passively viewing pictures with facial emotion expressions) with varying levels of ecological validity to assess individual differences. However, the hallmark of social interaction is the circular exchange of social signals between two or more individuals. Thus, on-line tasks that include feedback loops between two or more social partners would be more appropriate to understand social cognitive processes (c.f., Dziobek, 2012).

Economic exchange games might present one fruitful approach to analyze bidirectional social interaction and the potential underlying social-cognitive processes in BPD. In these games, monetary units are considered proxies of exchange of social signals. In order to make decisions and predict the counterpart's behavior, players have to make inferences about the partner's intentions. In support of this view, Sally and Hill (2006) showed that theory of mind performance is related to cooperation and fair behavior in economic exchange games. Given that economic games rarely involve face-to-face encounters of interaction partners, a further advantage of these games lies in the fact that deviant facial expression in BPD would not be a confounding factor.

Unoka et al. (2009) showed that BPD patients transferred smaller amount of money units when playing the role of investor in a multi-round trust game (for explanation of trust games see Camerer and Weigelt, 1988) compared to non-clinical controls. In line with this finding, King-Casas et al. (2008) applied a multi-round trust game with BPD patients as trustees and found that investment significantly decreased in later rounds. Further, BPD patients (as trustees) offered significantly less reparative money (so called coaxing) compared to non-clinical controls, to repair cooperation when investment was low (King-Casas et al., 2008). Neurofunctionally, this deficit was related to deviant activation in the anterior insula (King-Casas et al., 2008). In summary, the results of both studies argue for a lack of cooperation, which might be based on reduced trust in BPD patients: the patients might infer the intentions of the partners to be non-cooperative. In contrast, a recent economic game study by Franzen et al. (2011) found that patients with BPD correctly estimate the fairness of a social partner. Interestingly, in their combination of a multi-round trust game and simultaneous presentation of varying emotional facial expressions of the game partners, the authors reported that while non-clinical controls disregarded the emotional facial expression of the partners in order to judge fairness, the BPD patients did not. Franzen and colleagues interpreted these results as evidence for enhanced intention reading by the patients with BPD (Franzen et al., 2011).

In sum, although economic games clearly represent a promising method for the on-line assessment of social interaction, their exact contribution to elucidating social cognition in BPD requires further study.

## Treatment implications

The literature reviewed here has several potential implications for the treatment of BPD. Given that data point toward deficits in social cognition that are likely amplified by emotion dysregulation and arousal, psychotherapeutic interventions designed to improve emotion regulation might also affect social cognition (e.g., Dialectic Behavior Therapy (DBT); Linehan, 1993; Systems Training for Emotional Predictability and Problem Solving (STEPPS); Blum et al., 2008). This potential causal relationship needs to be explored in future empirical research with BPD patients.

Further, within the context of clinical work with BPD patients, the emotions and cognitions of the therapist should not be assumed to be accurately understood by the patient implicitly, but rather should be explicitly expressed. Additionally, ambiguous emotional expressions, potentially hampering social interactions in general, should be taken into account, and if necessary, they should be explicitly addressed in single and group psychotherapeutic settings. Moreover, the therapist should be aware that a BPD patient's evaluation of other people has a tendency to be malevolent and possibly generally more extreme.

In addition, psychotherapeutic interventions and trainings for enhancing social cognitive abilities should be integrated into the treatment of this patient group, with special respect to PTSD and traumatic experiences. Although different psychotherapeutic programs such as DBT, Transference Focused Psychotherapy, Schema Focused Therapy, Supportive Psychotherapy, and Mentalization-Based Therapy, which all address social cognition in their own ways, have proven effective in the treatment of BPD (de Groot et al., 2008), information on their capacities to improve specific aspects of social cognition is still lacking.

Aberrations of the opioid, oxytocin, and vasopressin system have been hypothesized to contribute to interpersonal disturbance in BPD and potentially to impaired social cognition in this population (New and Stanley, 2010; Stanley and Siever, 2010). Thus, pharmacological intervention might be explored as an additional approach to improve social cognition capacities (e.g., Simeon et al., 2011; but see Bartz et al., 2011).

In summary, patients with BPD display a specific pattern of disturbance in cognitive and emotional empathy and expression of social signals. Given the importance of social cognition on the part of both the sender and the recipient for maintaining stable interpersonal relationships and establishing therapeutic alliance, these deficits should be explored further in future studies.

### Conflict of interest statement

The authors declare that the research was conducted in the absence of any commercial or financial relationships that could be construed as a potential conflict of interest.

## Key Concept

Affective instability: High intensity of affective states, rapid shifting between affect-categories, high reactivity to social stimuli, slow return to emotional baseline.
Social signals: Consciously or unconsciously perceived and expressed signals during social interaction such as, facial expression, gesture, body posture, eye gaze, speech.
Social cognition: Sum of cognitive processes that allow humans to interact with one another, substantially depending upon the exchange of social signals.
Cognitive empathy: Ability to understand the mental states of others, i.e., their thoughts, desires, beliefs, intentions, and knowledge, also referred to as perspective taking, mentalizing, or theory of mind.
Emotional empathy: Ability to share the emotional state of another person, emotional reaction in an observer to the affective state of another individual.
